# The Control of Winsley Sanatorium

**Published:** 1913-07-12

**Authors:** 


### THE CONTROL OF WINSLEY SANATORIUM.

### Breach of Agreement or Confiscation?

We gather that considerable heart-burning is being caused in Wiltshire and the Bristol neighbourhood by suggested changes in the constitution and management of Winsley Sanatorium. Undoubtedly the position of all existing sanatoria for the labouring classes has been so much affected by the operation of the National Insurance Act that new methods of finance and administration are in many cases unavoidable. Care is necessary in such cases that nothing shall be done to prejudice the position of patients, subscribers, or staff; for it would be a thousand pities should the Act result in injustice to any voluntary workers who have borne the burden and heat of the day in stirring up the national conscience to the point of taking an interest in the tuberculosis problem.

### A Pioneer Institution.

Winsley Sanatorium is one such pioneer institution, for it was one of the earliest, as it is still one of the best-known, sanatoria for the industrial classes. Like the Benenden institution, its existence is primarily due to the activity of the National Association for the Prevention of Consumption, and to some local medical men in Bristol and the "three counties" of Gloucestershire, Wiltshire, and Somersetshire. The land and original buildings were provided by the donations of the charitable; between 1899 and 1912 over £21,000 was thus received on capital account, apart altogether from subscriptions for maintenance. As in other instances (to Londoners, Benenden Sanatorium is a familiar case in point), the upkeep of a big consumption sanatorium proved so expensive a matter that business relations were established with commercial firms, industrial societies, municipal authorities, and anyone else who desired to have first call on sanatorium beds by paying £70 apiece per annum for maintenance. Such bargains were, of course, good business for both sides. The payers scored by being relieved of all trouble in arranging for treatment and maintenance of any consumptives they might be responsible for, and also by paying nothing for interest on the large amount of capital invested in land and buildings. The sanatorium managers scored by being relieved of anxiety as to annual income. The original donors of the £21,000 and the annual subscribers were protected by allocating twelve beds exclusively to their nomination, each such nominee paying fifteen shillings a week during his or her stay in the sanatorium. Further, they were protected by the presence of thirty-three representatives on the governing body, a number afterwards reduced to thirteen.

Such was the position of Winsley Sanatorium up till quite recently. It will be seen that the privileges of those whose generosity rendered the institution possible in the first place, and of those who have subscribed annually, were restricted to the first call on twelve beds for such patients as could furnish fifteen shillings a week—more than half the actual cost of their maintenance. The developments of the last year or two have resulted in a much greater demand than ever before for the beds allocated to local authorities. Part of this demand is to be met by structural additions and part, apparently, by raiding seven out of the twelve beds hitherto reserved for nominees of donors and subscribers. A resolution to this effect was carried at the annual meeting by one vote. The majority, it is stated, was mainly composed of representatives of Bristol Corporation and other similar bodies, and the minority of various founders and subscribers, including some of the medical men to whose energy and initiative the whole sanatorium is due. It was pointed out by one of the latter during the discussion that the capital provided by charitable people towards Winsley Sanatorium (apart altogether from their annual subscriptions) would not only provide the land and buildings for ten beds, but also endow them with an income of £70 a year each, so that to restrict the privileges of donors to the call on five beds, at half cost, is not exactly liberal treatment. To meet this argument the majority adopted a resolution to reduce the sum demanded from the patients in these five beds from fifteen to ten shillings a week, a slight concession in the right direction.

### Control and Local Authorities.

So far, then, a case seems to have been made out by those whose generosity and energy created the Winsley Sanatorium, against the usurpation of control by the representatives of local authorities, including the Corporation of Bristol. The object of these latter bodies is fairly clear. Saddled with certain responsibilities under the Insurance Act, they naturally desire to save new burdens on the ratepayers as far as possible. By utilising such institutions as Winsley they can provide sanatorium treatment at a far smaller cost, both for capital outlay and for maintenance, than if they were to start from the beginning. They argue that Winsley and similar institutions were provided for the benefit of the labouring classes, most of whom are now brought within the scope of the Insurance Act; and that therefore there is no reason why some of the beds hitherto reserved for donors and subscribers should not be made available for Insurance Act purposes. The fatal objection to this plan is that by saving the ratepayers an enormous capital outlay it biasses their representatives so deeply that it ought not to be carried out by the votes of those representatives against the wishes of the original members. For thus it practically amounts to confiscation by local authorities of large sums of money collected for charitable purposes. If the donors and their representatives acquiesced in this application of their moneys, no one could object. But the fact is that some of them do strongly object, and were in fact defeated at the meeting by only one vote.

So much for the constitution of Winsley Sanatorium. There are, we are informed, other grievances against the policy of the management. It is stated that the medical arrangements are unsatisfactory. That there is only one resident medical officer for 76 patients, and that he is deprived of control and authority to such an extent as to impair the efficiency of the institution. Further, that the report of the Medical Advisory Board, in which this defect was mentioned, was sent back to them by the Chairman with a request that this paragraph should be omitted. And that, after the Medical Board refused to delete the criticisms in question, the report was mutilated by someone before it was put on the agenda paper of the annual meeting.

If these allegations are true—and they are made by one in whose veracity we have full confidence—the Chairman of the Committee must see to it that an explanation is offered. The criticisms which are being levelled in Bristol against the reduction of donors' privileges are matters of opinion, upon which different views are legitimate and arguable. But actions such as this unauthorised mutilation of the Medical Board's report are in quite a different category. Nothing but unequivocal condemnation can possibly be meted out to such high-handed and tyrannical actions; and we trust that it has already been realised that an error was committed and that steps will be taken to repair it and to prevent any possibility of its repetition.

